# Fatty Acid Synthase: An Emerging Target in Cancer

**DOI:** 10.3390/molecules25173935

**Published:** 2020-08-28

**Authors:** Chee Wai Fhu, Azhar Ali

**Affiliations:** Cancer Science Institute Singapore, National University of Singapore, Singapore 117599, Singapore

**Keywords:** fatty acid synthase, cancer, lipid metabolism

## Abstract

In recent years, lipid metabolism has garnered significant attention as it provides the necessary building blocks required to sustain tumor growth and serves as an alternative fuel source for ATP generation. Fatty acid synthase (FASN) functions as a central regulator of lipid metabolism and plays a critical role in the growth and survival of tumors with lipogenic phenotypes. Accumulating evidence has shown that it is capable of rewiring tumor cells for greater energy flexibility to attain their high energy requirements. This multi-enzyme protein is capable of modulating the function of subcellular organelles for optimal function under different conditions. Apart from lipid metabolism, FASN has functional roles in other cellular processes such as glycolysis and amino acid metabolism. These pivotal roles of FASN in lipid metabolism make it an attractive target in the clinic with several new inhibitors currently being tested in early clinical trials. This article aims to present the current evidence on the emergence of FASN as a target in human malignancies.

## 1. Introduction

Cancer hallmarks were introduced by Hanahan et al. in 2010 and these hallmarks encompass six basic tumor characteristics—which are self-sufficiency in growth signals, insensitivity to anti-growth signals, tissue invasion and metastasis, unlimited replication potential, sustain angiogenesis, and evading apoptosis [1]. The whole paradigm was revised the following year to include metabolic reprogramming after a monumental effort had been spent on cancer metabolism studies [2]. Cancer metabolism was initially proposed by Otto Hendrich Warburg, termed Warburg’s effect, describing glucose consumption through glycolysis by cancer cells for ATP generation allowing tumor cell survival under aerobic condition [3]. In recent years, numerous studies have unraveled the dynamics of cancer metabolism and the concept of metabolic plasticity or metabolic rewiring of cancer cells was subsequently introduced. Apart from glucose utilization, cancer cells undergo various oncogenic mutations or adaptations to allow utilization of a more diverse range of nutrients including fatty acids (FAs) and amino acids for tumor survival, metastasis and disease progression. These findings have led to renewed interests to elucidate the diverse roles of lipid metabolism in cancer. This minireview aims to present current knowledge on fatty acid synthase FASN, its roles in cancer cell biology, metabolic reprogramming, and also the current challenges of FASN-targeted therapy.

## 2. FASN in Normal Physiology

**Figure 1:**
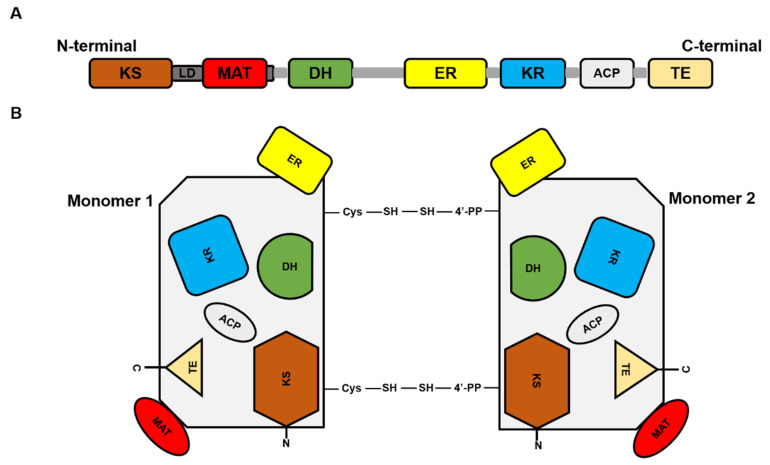
Fatty acid synthase (FASN) structure. (**A**) Represents the linear sequence organization of FASN monomer. (**B**). Structural overview of FASN comprising two identical monomers, each including seven catalytic domains: beta-ketoacyl synthase (KS), acetyl/malonyl transacylase (AT/MT), beta-hydroxyacyl dehydratase (DH), enoyl reductase (ER), beta-ketoacyl reductase (KR), acyl carrier protein (ACP), and thioesterase (TE).

FASN is a large multi-enzyme complex and the monomeric protein size is ~270 kDa. It comprises six separate enzymatic grooves that work together to produce a 16-carbon chain saturated fatty acid (FA), palmitate, from acetyl-coenzyme A (CoA) and malonyl-CoA in the presence of Nicotinamide adenine dinucleotide phosphate hydrogen (NADPH) [4]. The FASN monomer (Figure 1) possesses enzymatic activities which include beta-ketoacyl synthase (KS), acetyl/malonyl transacylase (AT/MT), beta-hydroxyacyl dehydratase (DH), enoyl reductase (ER), beta-ketoacyl reductase (KR), acyl carrier protein (ACP), and thioesterase (TE). Although the FASN monomer contains all the necessary enzymes needed for palmitate synthesis, the dimer formation is crucial for its function. The structure of FASN can be further categorized into three major domains where domain I contains KS, AT/MT and DH, domain II contains ER, KR and ACP, and domain III contains TE. About a quarter length of the monomer protein, located between domains I and II, which lacks catalytic activity, is called the interdomain/core region and is identified to be crucial for dimer formation [5].

FASN expression is critical for early embryo development, in which FASN knockout (KO) embryos fail to survive before implantation and the number of FASN heterozygous pups is 70% lower than predicted by Mendelian Inheritance, which indicate partial haploid insufficiency [6]. Furthermore, FASN expression is shown to participate in the proper development of the fetal lung and the normal functionality of the adult lung. There is ample evidence demonstrating that the fetal lung is capable of de novo FA synthesis and that FASN is required for surfactant production of alveolar epithelial cells [7]. After early development, FASN remains relatively quiescent in most tissues, however the reason why this is so still remains elusive. A plausible explanation is that non-actively proliferating tissues can meet the FAs’ demand from the diet to fulfil their physiological FA requirements. Nonetheless, a strong FASN expression has been reported in the lung, breast, liver, adipose and brain [8].

Deletion of FASN in alveolar type II epithelial cells is found to disrupt surfactant lipid composition and exacerbate injury response to bleomycin-induced fibrosis [9]. The mature mammary gland is a unique lipid metabolizing tissue where, in resting-state, it does not require fatty acid synthesis but strongly induces FASN during pregnancy and lactation [10]. De novo FA synthesis in the mammary gland is responsible for producing short and medium chain FAs in milk, which account for ~15–40% of total FA content [11,12]. Mammary gland-specific FASN KO mice are shown to suffer from growth reduction in mammary epithelial cells, alteration of the FAs profile in milk from lactating mothers, and also improper development of the functional lactating mammary gland [13].

FASN is considered as a housekeeping protein in the liver under normal physiological conditions where it controls the hepatic triglyceride mechanism. When carbohydrates are abundant, glucose are converted to FAs with the help of FASN. Excess FAs are then assembled into triglycerides and stored in the form of lipid droplets or secreted as very low-density lipoproteins [14,15]. During fasting or under glucose-depleted conditions, lipid droplets undergo lipolysis or catabolism and fatty acid oxidation to produce ketone bodies which are then used as fuel [16]. In the brain, FASN is essential to maintain proper development and control lipid metabolism in neural stem cells. Neural stem cells are responsible, not only for early brain development, but also remain active for an entire life to ensure proper brain function. They divide and generate new nerve cells to enable the brain to adapt to new arrangements. Furthermore, disruption of lipid metabolism in neural stem cells, through the expression of a non-functional FASN, results in learning and memory deficits in humans and mice [17].

## 3. FASN Regulation

FASN expression is regulated by external stimuli. A proposed mechanism for the upregulation of FASN transcription is through the activation of growth factor receptor (GFR) signaling pathways such as the epidermal growth factor receptor (EGFR), epidermal growth factor receptor 2 (HER2) and platelet-derived growth factor receptor (PDGFR) in tumor cells. The effects of GFR signaling on FASN regulation are complex and include cross-talk with other signal transduction pathways such as phosphatidylinositol-3′-kinase (PI3K) and extracellular regulated kinase 1/2 (ERK1/2). EGF activates EGFR activity, and together with ERK1/2 phosphorylation, they upregulate FASN in pancreatic ductal carcinoma (PDAC). Inhibition of EGFR by EGFR-specific siRNAs or EGFR-tyrosine kinase inhibitor (TKI) Erlotinib prevents FASN upregulation suggesting that EGFR activation is crucial for FASN upregulation [18]. Apart from EGFR, transcriptomic and protein analyses on HER2-amplified breast cancer cell lines showed enhanced FASN expression modulated through the PI3K-dependent pathway [19]. In a separate study, artificial amplification of HER2 cDNA in a non-tumor breast epithelial cell line, HB4a, forced the upregulation of lipogenic fatty acid translocase (CD36), fatty acid-binding protein 4 (FABP4) and FASN expression. Induction of lipogenesis in HER2 overexpressing cells was shown to be DEPTOR-mediated (DEP domain-containing mammalian target of rapamycin (mTOR)interacting protein), which inhibited the mTOR pathway [20]. Activation by PDGF promotes lipogenic phenotypes in fibroblasts and liver cancer cells. PDGF activation can phosphorylate PDGF-β receptors at tyrosine residues 740 and 751. Binding of PI3K to these phosphorylated sites is shown to elicit the upregulation of sterol regulatory element binding protein 1 (SREBP1), a positive regulator of FASN transcription. The importance of PDGF-β on PI3K activation is further supported by the failure to activate PI3K and lipogenic genes in HepG2 cells carrying mutated a PDGF-β receptor (at Y740/Y751) when compared to its wild-type counterpart [21].

Transcription of FASN can be regulated by multiple transcription factors. One of the most well-established transcription factors is SREBP. There are three isoforms of SREBPs: SREBP1a (encoded by the SREBF1 gene), SREBP1c (which arises from SREBF1 gene splicing) and SREBP2 (encoded by the SREBF2 gene) [22]. SREBP1c is involved in FA synthesis [23] and SREBP2 is relatively more specific to cholesterol synthesis [24]. SREBP1a is involved in both FA and cholesterol syntheses [25]. SREBPs levels are tightly regulated by sterol levels through a negative feedback loop mechanism. SREBPs are located at the endoplasmic reticulum (ER) membrane where they are associated with the SREBP cleavage-activating protein (SCAP) and the insulin-induced gene protein (Insig) when sterols are abundant. When sterol level drop, SCAP dissociates from Insig and transports SREBP to the Golgi where it undergoes cleavage at the N-terminus to generate the active form. Active cleaved SREBPs will then enter the nucleus and transcribe lipogenic genes such as FASN, by binding to the sterol regulatory elements within the gene promoter [26]. Although the activity of the PI3K/AKT and SREBP1c signaling cascade that regulates FASN expression is similar between normal and tumor cells, tumor FASN expression is found to be insensitive to nutrient levels as opposed to that in normal cells. This suggests the involvement of other oncogenic signaling pathways that may uncouple nutrient sensors from regulating FASN in tumor cells. This uncoupling mechanism may alter FASN function through posttranslational modification. In normal cells, SREBP1c is transcribed in response to nutrition and hormonal stimulation [27,28] whereas in cancer, it is regulated by aberrant growth factor levels such as the epidermal growth factor (EGF), platelet-derived growth factor (PDGF) or excessive steroid hormone signaling. In glioblastoma, EGFR-amplified tumors exhibit strong dependency on lipogenesis for growth. EGF induces the cleavage and AKT-mediated nuclear translocation of SREBP1c. The introduction of a constitutive activating EGFR mutant variant, EGFRvIII, in glioblastoma cells has been shown to exert a similar effect where tumor cells exhibit greater reliance on lipogenesis [29]. Steroid hormones such as androgen, progesterone and estrogen have been shown to be important in the development of prostate and breast cancers. Androgen-stimulated prostate cancer cells can proteolytically cleave SREBP1c and induce its nuclear translocation and FASN upregulation [30,31]. Furthermore, similar effects are seen in the breast cancer cell line, MCF-7, where progesterone stimulates both SREBP1c mRNA levels and protein expressions [32,33].

FASN protein stability may contribute to the elevated FASN levels seen in tumors, and this may provide an explanation on the lack of correlation between FASN mRNA and protein levels detected in tumors. FASN protein stability can be influenced by post-translational modification processes such as ubiquitination, sumoylation or acetylation, which can prevent FASN degradation. Malignant cells derived from different tissue types utilize distinct mechanisms to prevent or delay FASN degradation. In prostate cancer, the upregulation of isopeptidase ubiquitin-specific protease 2a (USP2a) after androgen stimulation, is shown to stabilize FASN expression by preventing ubiquitin-mediated degradation. FASN can interact with USP2a and it is shown that the catalytic core of USP2a possesses deubiquitinating activity in vitro. Binding of USP2a to FASN reduces its polyubiquitination thus slowing its degradation. The role of USP2a in FASN stabilization is verified by USP2a silencing or USP2a negative mutant (Δ276 Cysteine to Alanine) expression, which induces FASN degradation through the proteasomal degradation pathway [34]. In hepatocellular carcinoma, acetylation by histone K (lysine) acetyltransferase 8 (KAT8) destabilizes FASN by allowing FASN to interact with TRIM21 E3 ubiquitin-protein ligase for proteasomal degradation. However, elevated levels of deacetylated FASN have also been detected in hepatocellular carcinoma and this has been attributed to HDAC3 activity. Furthermore, a positive relationship has been established between these two proteins in clinical specimens [35].

Sumoylation is another form of FASN modification. In breast cancer cell lines, MCF-7 and SKBR3, Small ubiquitin-like modifier (SUMO)-conjugating enzyme Ubiquitin carrier protein 9 (UBC9) is shown to promote FASN sumoylation. In contrast to polyubiquitination, sumoylation promotes protein stability. Activity of UBC9 requires the co-operation of SUMO2, with the transfer of activated SUMO to FASN. SUMO2 is shown to protect FASN, where the combination of the protein synthesis inhibitor (cycloheximide) or proteasomal degradation inhibitor (MG132) with SUMO2 silencing, delays FASN degradation in breast cancer cell lines. Furthermore, the combination of SUMO2 silencing and cycloheximide treatment reduces FASN protein levels more than SUMO2 silencing alone while the inhibition of proteasomal degradation alone has no effect on FASN protein expression [36]. Acetylation can also influence FASN protein stability where an excessive amount of palmitic acid can upregulate acetyl-CoA acetyltransferase 1 (ACAT1), which in turn acetylates glyceronephosphate o-acyltransferase (GNPAT) and represses tripartite motif-containing protein 21 (TRIM21)-mediated FASN degradation during liver tumor progression [37].

In addition to proteasome degradation, autophagy can influence FASN degradation. In acute myeloid leukemia (AML), FASN expression is found to be significantly higher in the AML cohort when compared to granulocytes and CD34+ hematopoietic progenitor cells from healthy donors. The elevated FASN expression in AML is linked to impaired autophagy where FASN escapes autophagic degradation in AML through mTOR pathway activation. FASN expression enhances mTOR activity leading to autophagy related 1 (ATG1) phosphorylation at Serine 757 and this reduces ATG1 activity which affects its autophagic capability. ATG1 is a key autophagic protein in the initiation complex. Furthermore, FASN expression can negatively regulate transcription factor EB (TFEB) through the mTOR pathway. Activated mTOR can phosphorylate TFEB leading to its sequestration within the cytoplasm and inhibit its transcriptional activity. TFEB is a key transcriptional regulator of more than 500 genes comprising the CLEAR (Coordinated Lysosomal Expression and Regulation) network of autophagy and lysosomal genes. FASN inhibition is shown to promote TFEB nucleus translocation and enhance lysosome biogenesis [38].

FASN has been shown to auto-regulate its expression through cross-talk with the PI3K/AKT pathway. In human osteosarcomas, a positive correlation between PI3K/AKT activation and FASN overexpression is observed in a high proportion of clinical specimens. FASN suppression is shown to reduce AKT phosphorylation and, conversely, AKT inhibition demonstrates a similar downregulation effect on FASN mRNA and protein expressions in vitro [39]. Apart from the PI3K/AKT pathway, a similar observation can be seen in breast cancer cells, which identifies leukotriene B4 (LTB4) as the downstream product of 5-lipoxygenase (5-LOX), that induces FASN expression. FASN suppression is shown to downregulate ERK 1/2 phosphorylation and 5-LOX expression, which forms a positive ERK/LOX/LTB4 feedback loop mechanism [40].

## 4. FASN and Cancer

The fatty acid (FA) is an essential molecule in the entire lipid metabolism. It is responsible for the assembly of all biological membranes, precursors for secondary messengers, and is an important substrate for higher ATP production. A cancer cell derives FA from two main sources. It can obtain free FA either exogenously from the microenvironment or endogenously through de novo synthesis by FASN. In terms of FA uptake, cancer cells are equipped with several specialized transporters to facilitate FA movement across membrane bilayer. The most well characterized FA transporters include CD36, solute carrier protein family 27 (SCL27) and fatty acid binding proteins (FABPs). CD36 can transport long chain fatty acid [41], oxidized- low density lipoprotein [42], anionic phospholipids [43] and oxidized phospholipids [44] across the cellular membrane. CD36 expression is found to be significantly upregulated in malignant tissues including ovarian [45], gastric [46], breast [47] and hepatocellular carcinoma [48]. Furthermore, its expression profile is highly associated with the disease stage and metastatic status [49]. SLC27 consists of a family of six members, from SLC27A1 through SLC27A6, for the uptake of long chain fatty acids. Each family member displays specific substrate and tissue distribution, and the expression of SLC27 family members is associated with tumor fatty acid uptake [50,51,52,53]. FABPs are a group of low molecular weight proteins (14–15 kda) comprising 12 family members. Each FABP possesses a unique pattern of tissue expression and that a particular tissue type can concurrently express several types of FABP. FABP acts as a lipid chaperone that binds to both saturated and unsaturated long chain FAs, and other hydrophobic ligands such as eicosanoids, monoacylglycerols and endocannabinoids [54,55,56,57]. FABPs can facilitate lipid transportation to various cellular organelles including mitochondria, peroxisomes and the nuclei [58]. FABPs are frequently found upregulated in various malignancies including prostate, bladder and renal cell carcinoma [59,60,61].

There is a clear difference in lipid metabolism between normal and tumor cells. Normal cells derive FAs exogenously while tumor cells derive FAs both exogenously and by de novo synthesis through FASN. Tumors or precursor lesions undergo excessive de novo FA synthesis irrespective of circulating lipid levels. In contrast to normal cells, almost all triacylglycerol FAs in tumor cells are derived from de novo synthesis [62]. Though de novo synthesis of FA and glycolysis in cancer are closely associated with elevated lipogenic and glycolytic enzymes activities [63], lipogenesis in cancer did not attract much interest among cancer biologists until the mid-1990s. Renewed interests in lipid metabolism started after the discovery of FASN, formerly known as oncogenic antigen-519, in breast cancer in 1994 [64]. Eukaryotes harbor two distinct FA synthesis systems [65]. FASN exists exclusively in the cellular cytoplasmic compartment and is referred to as the type I fatty acid synthesis system while the type II fatty acid synthesis system is present in mitochondria and termed mitochondrial FAS. Both systems produce two distinct products.

Mitochondrial fatty acid synthesis is functionally different from cytoplasmic fatty acid synthesis as it does not contribute significantly to cellular triglyceride storage or phospholipids. Furthermore, products from mitochondrial FAS cannot be substituted by delivery of FAs from extra-mitochondrial origin. The main product of mitochondrial FAS is lipoic acid, which is an important lipid co-factor for multiple mitochondrial dehydrogenases and is crucial for optimal mitochondrial function [66]. Up until now, little is known on mitochondrial FAS and its association with carcinogenesis. Extensive studies have focused on the cytoplasmic FA synthesis system, which is a type I FA system or FASN, and have sparked huge interests to investigate the roles of FASN in cancer. Like other proteins, FASN’s function is greatly affected by its localization, post-translational modification and protein–protein association. Although FASN has been frequently described as a cytoplasmic protein, it can also be found to localize in other intracellular compartments such as the nucleus [67] and membrane of peroxisomes [68].

FASN can be modified through phosphorylation by protein kinases such as mammalian target of rapamycin (mTOR) and human epidermal growth factor receptor 2 (HER2), which can greatly impact its activity. In the liver, FASN can be found in the membrane and cytoplasm. When mice are fed with a normal diet, cytoplasmic FASN is preferentially phosphorylated at Threonine-1029 (T1029) and Threonine-1033 (T1033) compared to in a fasting state. This leads to downregulation of cytoplasmic FASN activity and reduces the activity of downstream proteins such as peroxisome proliferator-activated receptor α (PPARα). In the liver cell line Hepa1-6, treatment with mTORC1 inhibitor prevents phosphorylation of FASN at T1029 and T1033 residues, and restores its activity [69]. In SKBR3, a HER2 overexpressing breast cancer cell line, heregulin stimulation promotes phosphorylation and heterodimerization of HER2 leading to FASN phosphorylation. Phosphorylated FASN then forms a complex with HER2 that enhances FASN activity by increasing intracellular lipid content. Disruption of HER2 heterodimerization by heregulin is shown to dissociate FASN from the HER2 complex, which in turn reduces FASN phosphorylation and loss in FASN activity [70].

FASN can also form complexes with other proteins such as caveolin-1 and protrudin to expand its functions. In prostate cancer, FASN and caveolin-1 are coordinately expressed where the levels of both proteins are seen increasing from normal to malignant state, and that the increment magnitude is in line with tumor progression. FASN can co-immunoprecipitate with caveolin-1 and this association is dependent on the palmitoylation of caveolin-1 at Cys-156. Site-directed mutagenesis at Cys-156 prevents caveolin-1 palmitoylation and deters the association between FASN and caveolin-1. This signaling axis is an important signal transduction pathway to ensure heightened and continuous phosphorylation of AKT and proto-oncogene tyrosine-protein kinase sarcoma (Src) in tumors [71]. Membrane protrusion formation capacity is a key feature in developing neurons and many eukaryotic cells. This phenotype involves active cytoskeleton remodeling mediated by protrudin. In HeLa cells, protrudin transiently interacts with FASN and this interaction is dependent on the presence of free FAs. Surprisingly, FASN and protrudin expressions positively regulate one another where FASN suppression results in reduced protrudin expression and vice versa [72].

The idea of a metabolically homogenous cancer cell population, in which cancer cells are shown to be metabolically heterogeneous with one subpopulation depending on glycolysis while another subpopulation is entirely dependent on oxidative phosphorylation to survive and thrive, has been challenged [73]. Furthermore, different stages of cancers possess different energy requirements for progression. Metastatic cancer cells require high amounts of ATP and predominantly rely on the mitochondria’s oxidative phosphorylation to generate sufficient energy [74]. Furthermore, different organelles have been shown to utilize FAs differently to maintain homeostasis and optimal function suggesting FASN’s influence on an organelle’s function which affects cellular metabolism. Table 1 shows a summary on the roles of FASN reported in various malignancies.

## 5. FASN Influences Organelle Activity in Tumor Cells

### 5.1. Mitochondria

Mitochondria are responsible for oxidative phosphorylation and are the powerhouse of the cell. Mitochondria functions are affected by three main factors—mitochondrial biogenesis, mitochondrial dynamics and types of substrate utilized in mitochondrial respiration. FAs can be broken down to generate a high energy molecule, acetyl-CoA, which then feeds into mitochondria for higher ATP production. Nonetheless, the impact of FASN overexpression on mitochondria respiration had not been well studied until 2015. A positive association between FASN levels and oxidative phosphorylation rates has been reported across various cancer cell types and immune cells through the manipulation of FASN levels [77,90]. FASN overexpression is shown to generate excessive amounts of free FAs, which are then broken down into acetyl-CoA. Acetyl-CoA then supports mitochondria respiration through fatty acid oxidation (FAO) to generate a greater amount of energy compared to glucose. Due to the dynamic nature of cancer metabolism, abundance levels of fatty acids in cells allow them to be an indispensable fuel source for cellular respiration, particularly in triple-negative breast cancer [91]. Furthermore, FAO can lead to higher levels of ATP production and promote cancer metastasis which demands greater energy [78]. In an in vitro colorectal cancer model, FASN inhibition is shown to lower mitochondrial respiration and FAO. Supplementation of nutrients such as glucose and glutamine in the culture medium fails to rescue mitochondria respiration and FAO in FASN-suppressed cells. This highlights the control that FASN has over FAO levels under conditions of different substrate availability. Furthermore, the elevated mitochondrial respiration and fatty acid oxidation link a positive association between FASN and the high energy demand metastatic cancer cells [78,79,80]. The role of FASN in metabolic rewiring is demonstrated in a recent study where FASN overexpression in senescent cells induces mitochondria respiration and is accompanied by an elevation of mitochondria biogenesis. In addition, a significant increase in cytochrome c oxidase subunit 4 isoform 1, mitochondrial (COXIV) (a mitochondria-specific marker) and peroxisome proliferator-activated receptor gamma coactivator 1-alpha (PGC-1α), as well as a higher mitochondria mass is shown in FASN overexpressing senescent cells and these changes can be reversed with the FASN inhibitor, C75 [92].

The mitochondrion is a dynamic organelle and the frequent cycles of fusion and fission, with the help of specific proteins, allow for the adaptation to changes in the metabolic needs of a cell. Mitochondria undergo an extensive fusion process and form hyperfused networks in respiratory active cells. Dynamin-related guanosine triphosphate hydrolases (GTPases), such as dynamin-1-like protein (Drp-1), mitofusin 1 (Mfn1), mitofusin 2 (Mfn2) and optic atrophy 1 (OPA-1), are crucial in the regulation of mitochondria dynamics. Translocation of Drp-1 from the cytosol to mitochondria promotes mitochondrial fission while OPA-1 is required for the fusion of the inner mitochondria membrane [93]. In pancreatic and breast cancer cell line models, EGFR overexpressing cancer cells are shown to contain two distinct subsets of EGFR localization, which are plasma EGFR (pmEGFR) and mitochondria EGFR (mtEGFR). pmEGFR is shown to interact with FASN forming a complex, and this association is found to be independent of EGFR kinase activity. However, FASN phosphorylation, upon EGF stimulation, relies on pmEGFR kinase activity and can be inhibited by an EGFR kinase inhibitor. FASN phosphorylation can promote de novo palmitate synthesis. Following an increase in palmitate production, mtEGFR is palmitoylated at cysteine residues 781 and 797. mtEGFR palmitoylation can induce its phosphorylation and activity, similarly to the induction by EGF treatment. The activated mtEGFR then induces prohibitin 2 (PHB2) and OPA-1 expressions without affecting the expression of mitochondria fusion proteins, Mfn1 and Mfn2. Blocking the palmitoylation process with an FASN inhibitor, cerulenin, reduces mtEGFR phosphorylation and mitochondrial fusion suggesting that palmitic acid, synthesized by FASN, regulates palmitoylation and mtEGFR activation to promote the mitochondria fusion process [81].

### 5.2. Peroxisomes

Peroxisomes are small, spherical and single membrane organelles found in most eukaryotic cells. Most of the biochemical pathways in peroxisomes involve β-oxidation of fatty acids, α-oxidation of branched-chain fatty acids and cholesterol metabolism [94]. Peroxisome proliferator-activated receptors (PPARs) were originally identified in Xenopus frogs as receptors that regulate the proliferation of peroxisomes in cells [95]. Subsequent studies then showed that PPARs are nuclear receptors and function as molecular FA sensors in homeostatic energy regulation of cancer cells. Three isoforms of PPARs have been identified namely PPARα, PPARβ/δ and PPARγ, and each isoform is activated by specific ligands and possesses tissue-specific expression profiles. Under normal physiological conditions, cross-talk between FASN and PPARα forms a complex network that regulates energy homeostasis in the brain and liver [96,97]. A positive correlation is shown to exist between PPARα and FASN activities and this is largely due to the formation of PPARα ligands, derived from FAs during lipolysis, lipogenesis or FA catabolism [98]. PPARα is a transcriptional factor and plays important roles in the regulation of peroxisomal and mitochondrial β-oxidation, and FA transport genes [99,100]. However, in cancer, the functional relationship between FASN and PPARα is controversial and it differs between cancer types. In breast cancer, FASN–PPARα functions as a negative feedback loop axis where PPARα activation is shown to suppress FASN expression through SREBP-1c inhibition [82]. Additionally, PPARα activation can induce carnitine palmitoyltransferase 1a (CPT-1a) expression, a gene that mediates fatty acid oxidation, thus demonstrating a metabolic shift from the lipogenic to lipolysis phenotype. In contrast, PPARα is shown to induce FASN, mitochondrial fatty acid elongation and fatty acid metabolism genes transcriptionally, triggered by 4-(methylnitrosamino)-l-(3-pyridyl)-ibutanone (NNK) in a mouse lung cancer model [101]. To date, there is no clear explanation for the differences in PPARα’s function between different tissue types. Furthermore, PPARα is capable of forming various transcriptional complexes with different proteins such as retinoid X receptors (RXR) and PGC-1α to regulate different lipid metabolism genes. The signaling axis partnership formed between activated PPARα and PGC-1α is also shown to regulate fatty acid transport and oxidation [102].

### 5.3. Nucleus

The influence of FASN on nucleus function is relatively unknown, although limited studies have shown that FASN is critical in mediating DNA damage response in cancer cells. Overexpression of FASN is shown to upregulate non-homologous end-joining (NHEJ) activity and repair DNA damage in MCF-7 and Panc-1 cells upon genotoxic insult. FASN is shown to suppress nuclear factor kappa-light-chain-enhancer of activated B cells (NF-kB), nuclear factor NF-kappa-B p65 subunit (p65) expression and induce specificity protein 1 (SP1) protein expression and leads to the upregulation of poly adenosine diphosphate (ADP)-ribose polymerase 1 (PARP-1). Enhanced PARP-1 expression, in turn, promotes Ku protein recruitment and DNA repair. Moreover, lipid deprivation suppresses SP1 expression and reduces NHEJ DNA damage response and can be rescued by palmitate supplementation. However, lipid deprivation or palmitate supplementation has no effect on NF-kB expression, suggesting that FASN regulation of NF-kB and SP1 expressions occurs through different mechanisms [83]. In addition, lentiviral-mediated FASN inhibition can enhance radio-sensitivity of non-small cell lung cancer (NSCLC) cells through suppressing the double-strand break DNA damage response. DNA damage response suppression occurs through the inhibition of the DNA-dependent protein kinase catalytic subunits (DNA-PKcs), key proteins associated with DNA double strand break repair, and prevent its increase following radiation [86]. The nuclear envelope comprises two lipid bilayers containing phosphotidylcholine, sphingomyelin, fatty acids and eicosanoids [103]. De novo FA synthesis is required for nuclear envelope assembly and successful mitosis. Extensive metabolic changes have been observed to occur during cell cycle processes with notable increases in phospholipids and fatty acids levels. As cells progress towards the Synthesis (S) and Growth 2/Mitosis (G2/M) phase, de novo FA synthesis activity is elevated significantly. FASN activity inhibition by C75 induces cell cycle arrest at G2/M and is accompanied with cyclin B1 upregulation. Furthermore, exogenous palmitate fails to reverse the inhibitory effect, suggesting that endogenously synthesized FAs are critical for completion of the cell cycle [104]. In a follow-up study, upregulation of endogenous FA synthesis is shown to be directed towards the nuclear envelope at the mitotic exit [105]. Nuclear localization of FASN was first reported in 2014 where it was shown to correlate with disease aggressiveness in prostate cancer [67]. Due to the scarcity of information on nuclear FASN, future studies are warranted to elucidate its role as a nuclear protein and identify its interacting partners in malignant cells.

### 5.4. Endoplasmic Reticulum

**Figure 2:**
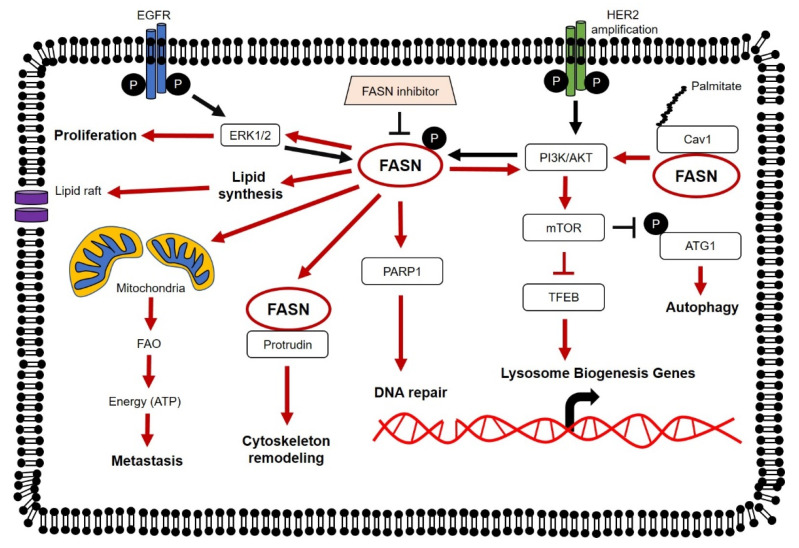
Molecular mechanisms of FASN in cancer. Expression and activity of FASN can be regulated by the epidermal growth factor receptor (EGFR) family members, EGFR and epidermal growth factor receptor 2 (HER2). FASN is shown to regulate lipid synthesis, signaling of major oncogenic pathways (including phosphatidylinositol-3′-kinase (PI3K/AKT) and extracellular regulated kinase 1/2 (ERK1/2)) and modulate cellular mechanisms (including autophagy, DNA repair and transcription of Lysosome Biogenesis genes). FASN overexpression promotes proliferation and increases the metastatic potential of tumor cells.

The endoplasmic reticulum (ER) is an organelle comprising two subunits, rough and smooth ER. This organelle is responsible for proper protein folding and transport of secretory and membrane proteins. Disruption of ER homeostasis leads to the accumulation of misfolded and unfolded proteins within the ER lumen leading to ER stress. FASN inhibition has been shown to induced ER stress in HT-29, a colorectal cancer cell line. In HT-29 cells, FASN inhibition induces a robust protein kinase R (PKR)-like endoplasmic reticulum kinase (PERK)-dependent phosphorylation of the translation initiation factor, eukaryotic translation initiation factor 2α (eIF2α), and concomitant protein synthesis reduction. Furthermore, pharmacological inhibition of FASN is shown to upregulate processing of X-binding protein-1 (XBP-1) and the inositol-requiring enzyme 1 (IRE1) arm of the ER stress, which then induces the activation of the unfolded protein response (UPR) pathway [87]. There is limited knowledge on the impact of ER-stress on lipid metabolism, particularly between FASN and ER in cancers. However, scant evidence from several reports demonstrates that ER stress plays a crucial role in maintaining lipid metabolism homeostasis, particularly in the liver. UPR pathway activation, induced by ER stress, suppresses the expression of transcription factors required to control key lipid metabolic genes [106]. An unexpected direct link has been reported between ER homeostasis and transcription regulation of metabolic genes which suggests ER stress as the underlying factor behind fatty liver disease. In this study, the authors showed that activating transcription factor 6 (ATF6) knock-out mice exhibited greater susceptibility to ER stress and persistence of ER stress triggered the downregulation of several key mediators of lipid metabolism genes such as FASN, SREBP1, PPARα and PGC-1α. Deletion of ATF6, however, failed to re-sensitize cells to ER stress but instead rendered them incapable of recovery or adapting to ongoing stress. Using eIF2α and PERK transgenic mice, they further demonstrated that the suppression of lipid metabolic genes by ER stress was independent of any specific UPR pathway [107]. From this gathered information, we postulate that FASN is necessary to maintain ER homeostasis and proper functioning of ER is needed for optimal lipid metabolic gene expression such as FASN. However, this assumption needs to be tested further with more detailed studies. An overall view of the molecular mechanisms of FASN in cancer is illustrated in Figure 2.

## 6. FASN Is Functionally Linked to Metabolic Pathways

Metabolic pathways in a cancer cell are interconnected. Products of glycolysis and pyruvate metabolism are converted into substrates for lipid synthesis and amino acids are converted into substrates in Kreb’s cycle and mitochondrial respiration. This highlights the flexibility of cancer cells to survive under adverse conditions. FASN does not only affect lipid metabolism but it can also affect glycolysis. In colorectal cancer cell lines, suppression of FASN expression is shown to impair glycolytic capacity and reserves of HT29 and HCT116 cells. It has been suggested that glycolytic pathway inhibition is linked to lipid synthesis-derived NADP+, which increases the cytosolic pool of NADP+ to maintain glycolysis activity and downregulates mitochondrial-bound hexokinase activity [77]. Similarly, FASN inhibition in breast cancer and non-Hodgkin lymphoma cell lines exhibits impaired glycolysis process. In breast cancer cell lines, HER2 is activated by heregulin-β1 (HRG-β1) to induce glycolysis in high FASN and HER2 expressing SKBR-3 cells. FASN inhibition with cerulenin in SKBR-3 cells leads to a significant reduction in glucose uptake and lactate production. HER2 overexpression in low FASN and HER2 expressing MCF-7 cells, however, can induce FASN expression and glycolysis after HRG-β1 stimulation. Subsequently, loss of FASN activity abrogates these observations, irrespective of HER2 activation, indicating a functional link between FASN and glycolysis [84]. In non-Hodgkin lymphoma cell lines, cross-talk between glycolysis and FASN-mediated lipid synthesis is shown to be PI3K/AKT pathway-dependent, in which inhibition of FASN activity impairs cellular glycolytic flux and vice versa [85].

Apart from glycolysis, FASN is shown to be functionally linked to other metabolic pathways in NSCLC cells with low glycolytic activity. Suppression of FASN activity in these NSCLC cells induces multiple adaptive changes in FA synthesis and other associated metabolic pathways, including ketone metabolism and glutaminolysis. These metabolic changes, detected through radioisotope-labeling of metabolites, are concomitant with changes seen in the expression of specific metabolic genes [108]. A genome-wide study on the breast cancer cell line, MDA-MB-435, also reiterates the ability of FASN to regulate various metabolic pathways. Using Gene Set Enrichment Analysis, FASN activity inhibition is shown to induce widespread changes in tumor metabolism in which FASN inactivation downregulates glycolysis/gluconeogenesis and the Krebs cycle pathway. Furthermore, downregulation of the Krebs cycle pathway is consistent with overall suppression of mitochondrial genes involved in energy metabolism and oxidative phosphorylation [109].

## 7. Targeting FASN in Cancer

In recent years, there has been a resurgence in interests for the development of lipid inhibitors in anti-cancer therapy (Table 2) [110]. Distinct FASN activity or expression between normal tissues and cancer cells can be exploited and several FASN specific inhibitors have been developed which include cerulenin, C75, orlistat and the recently developed TVB-2640. Pharmacological inhibition of FASN has been shown to be effective in various malignant cells in vitro and in vivo but not in normal cells, and this presents a therapeutic window for intervention [4]. Cerulenin and C75 are early small molecule FASN inhibitors. Cerulenin, isolated from Cephalosporium caerulens, contains an epoxy group that reacts with the ketoacyl synthase domain of FASN. It was one of the first FASN inhibitors that showed anti-cancer effects in breast and ovarian cancer both in vitro and in vivo. Treatment of breast cancer cell lines, SKBR3 and MCF-7, with cerulenin inhibits FASN activity, reduces clonogenic properties and induces programmed cell death [88]. Cerulenin treatment of mice carrying ovarian cancer OVCAR-3 xenografts shows a promising anticancer effect where tumor FASN activity is greatly reduced and is accompanied by regression of established ascites tumors with significant improvement in mice survival at end-point [111]. Though cerulenin displays promising therapeutic efficacy against various cancer types both in vitro and in vivo, the highly reactive nature of cysteine reactive epoxide groups and off-target activities prevent its clinical development [112]. To circumvent this problem, C75 is developed based on a cerulenin binding mode and it can interact with FASN domains containing three different enzymatic functions including TE, KS and ER where strong anti-tumor activity is observed both in vitro and in vivo [113,114]. However, like cerulenin, C75 shows side effects including severe weight loss and profound changes in food intake in mice [115], and non-specific binding to other proteins like CPT-1 [116] and glyceraldehyde 3-phosphate dehydrogenase (GAPDH) [117], which affects its clinical development.

Another FASN inhibitor that is being extensively tested is Orlistat. Orlistat is a reduced form of the natural product, lipstatin, isolated from Actinobacterium Streptomyces toxytricini. The intended use of Orlistat is for obesity treatment. It acts by binding irreversibly and inhibits the TE domain of FASN. Orlistat’s mechanism of action in preventing obesity is through blockage of free FA absorption in the gastrointestinal tract by inhibiting pancreatic and gastric lipase [118]. Orlistat contains a highly reactive beta-lactone that covalently captures reactive serine residues such as Ser2308 within the TE domain of FASN [119]. Its anti-tumor properties have been explored extensively where studies have shown shrinkage in tumors both in vitro and in vivo [75,89]. However, Orlistat is highly unstable due to the presence of beta-lactone, has poor water solubility and poor gastrointestinal absorption that hinders its advancement in clinical trials [120].

A better understanding of the FASN structure has since led to the development of more specific FASN inhibitors with less downsides. One of the most promising candidates is TVB-2640, an oral, once daily dosed inhibitor. TVB-2640 demonstrates prolonged stable disease when given as monotherapy and confirmed partial response in Response Evaluation Criteria in Solid Tumors (RECIST) when combined with paclitaxel. Responses were seen across various tumor types including Kirsten RAt Sarcoma virus (KRAS) mutated non-small cell lung cancer, ovarian and breast cancers [121]. TVB-2640 is currently being evaluated in a Phase II clinical trial (NCT03179904), in combination with trastuzumab and paclitaxel, in late stage of HER2+ breast cancers.

Not all tumor cells are adversely affected by FASN inhibition. Identifying susceptible cancer cell populations resistant to FASN inhibitors is therefore critical. In pancreatic cancer, metabolite profiling of pancreatic ductal adenocarcinoma (PDAC) has led to the identification of three subpopulations associated with glycolysis, lipolysis and redox balance within the same tumor population. Each subtype displays different metabolic properties and sensitivity to different classes of metabolite inhibitors. Glycolytic cells are sensitive to glycolysis inhibitors whereas FASN inhibitors have no effect on these cells. In contrast, lipogenic cells are generally sensitive to FASN inhibitors although there are several lipogenic cell types that remain unaffected. These observations probably arise due to the metabolic plasticity of cancer cells that undergo additional adaptation stages against FASN inhibitors [122]. Likewise, treatment of colorectal cancer with a different FASN inhibitor, TVB-3166, showed similar observations. In a panel of colorectal cancer cell lines and primary colorectal cancer cells, TVB-3166 has shown a promising anti-tumor response that correlates with reduced FASN levels. However, tumors from patient-derived xenograft (PDX) models demonstrate a greater variation in response to TVB-3166 without a clear association between FASN expression and drug response. A detailed screening of PDX tumors reveals that sensitivity to TVB-3166 is influenced by Kirsten RAt Sarcoma (K-Ras), tumor protein p53 (TP53) mutation, 5’ adenosine monophosphate-activated protein kinase (AMPK) and AKT pathway activation, and stored lipid content (using TIP47 as a lipid marker) [76]. Together, these lines of evidence suggest that FASN levels are an important selection criterion for FASN-targeted therapy, however not all FASN-positive tumors may respond to therapy.

## 8. Conclusions

Lipid metabolism is now recognized as an important pathway in cancers. Lipid metabolism can provide additional sources of energy required for metastasis, assembly blocks for proliferation and act as secondary messengers in various signaling pathways. FASN is an essential molecule in the lipid metabolic pathway and is capable of rewiring tumor cells for greater energy flexibility to attain their high energy requirements. Studies have revealed that, apart from lipid metabolism, FASN has functional roles in other processes such as glycolysis and amino acid metabolism. The pivotal role of FASN in lipid metabolism makes it an attractive target in the clinic with the FASN inhibitor TVB-2640, currently in Phase II clinical trials, showing the greatest potential of progressing towards the clinic. However, undesirable extensive cross-talk between FASN and metabolic/oncogenic pathways may dampen its success in the clinic. Furthermore, the identification of FASN-positive tumor signatures can serve as predictive biomarkers to select cancer patients who will likely to benefit from FASN-targeted therapy. More importantly, further investigation is warranted to gain a better understanding of the relationship and dynamics between cancer metabolism, oncogenic mutations and activation of other signaling pathways in FASN-positive tumors.

## Figures and Tables

**Table 1:** Roles of FASN in various malignancies.

Role of FASN	Function	Test Model	Tumor Type	Reference
Promote	Lipogenesis	In vitro	Breast	[70]
	Signal transduction	In vitro, Mouse	Breast, Prostate, Colorectal, Non-small Lung	[40,71,75,76]
	Cytoskeleton remodeling	In vitro	Cervical	[72]
	Mitochondria respiration	In vitro, Mouse	Colorectal, Melanoma, Breast	[77,78,79]
	Fatty Acid Oxidation	In vitro, Mouse	Melanoma, Breast, Colorectal	[78,79,80]
	Mitochondria fusion	In vitro	Prostate, Breast	[81]
	Lipolysis	In vitro	Breast	[82]
	DNA repair	In vitro	Breast, Pancreatic	[83]
	Cell cycle	In vitro	Cervical	[9]
	Glycolysis	In vitro	Colorectal, Breast, Non-Hodgkin Lymphoma	[77,84,85]
	Cell migration	In vitro	Breast	[84]
Repress	DNA repair	In vitro	Non-small Lung	[86]
	Unfolded Protein Response	In vitro	Colorectal	[87]
	Programmed cell death	In vitro	Breast, Pancreatic	[88,89]

**Table 2:** Summary of FASN inhibitors tested against various malignancies.

Inhibitor	Drug Development Stage	Test Model	Disease Type	Reference
Cerulenin	Preclinical	In vitro	Breast Cancer	[88,123,124]
			Colon Cancer	[125]
			Adenocarcinoma Lung Cancer	[126]
			Colorectal Cancer	[127]
			Retinoblastoma	[128]
			Ocular Cancer	[129]
			Bladder Cancer	[130]
			Melanoma	[131]
		Mouse	Ovarian Cancer	[111]
			Adenocarcinoma Lung Cancer	[126]
C75	Preclinical	In vitro	Breast Cancer	[113,132,133,134,135]
			Adenocarcinoma Lung Cancer	[114]
			Gastric Cancer	[136]
			Endometrial Cancer	[137]
			B-cell Lymphoma	[138]
		Mouse	Adenocarcinoma Lung Cancer	[114]
			Prostate Cancer	[139]
Orlistat	FDA Approved	Human	Obesity Management	[118]
	Preclinical	In vitro	Non-Small Cell Lung Cancer	[75,140]
			B-cell Lymphoid Cancer	[141]
			Pancreatic Cancer	[89]
			T-cell Leukemia	[142]
			Prostate Cancer	[143,144]
			Melanoma	[131,145]
			Retinoblastoma	[128]
			Hepatocellular Carcinoma	[146]
			Oral Squamous Cell Carcinoma	[147]
			Ovarian Cancer	[148]
			Head and Neck Squamous Cell Cancer	[149]
			Brain Cancer	[150]
		Mouse	Gastrointestinal Cancer	[151]
			Oral Squamous Cell Cancer	[148]
			T-cell Lymphoma	[152,153]
			Ovarian Cancer	[149]
			Colorectal Cancer	[154]
			Melanoma	[145,155]
			Prostate Cancer	[143]
			Non-Small Cell Lung Cancer	[75,140]
TVB-2640	Phase 1 Clinical Trial (ongoing)	Human	Colon Cancer and other cancer type that can be removed by surgery (ID: NCT02980029)	[156]
	Phase II Clinical Trial (ongoing)	Human	Breast Cancer (ID: NCT03179904)	[156]
			KRAS mutated Non-Small Cell Lung Cancer (ID: NCT03808558)	[156]
			Astrocytoma (ID: NCT03032484)	[156]
TVB-3664	Preclinical	In vitro	Colon Cancer	[76]
		Mouse	Colon Cancer	[76]
TVB-3166	Preclinical	In vitro	Oral Squamous Cell Cancer	[157]
			Bladder Cancer	[158]
			Colorectal Cancer	[4]
			Non-Small Cell Lung Cancer	[4]
			Prostate Cancer	[4]
			Breast Cancer	[4]
			Ovarian Cancer	[4]
		Mouse	Pancreatic Cancer	[4]
			Ovarian Cancer	[4]
			Non-Small Cell Lung Cancer	[4]
